# Impact of the socioeconomic crisis in Lebanon on people with epilepsy

**DOI:** 10.1002/epi4.13084

**Published:** 2024-10-26

**Authors:** Joseph N. Samaha, Jim B. Dagher, Karine J. Abou Khaled

**Affiliations:** ^1^ Department of Medicine, Hotel‐Dieu de France Hospital Faculty of Medicine, Saint‐Joseph University Beirut Lebanon; ^2^ Department of Neurology, Hotel‐Dieu de France Hospital Faculty of Medicine, Saint‐Joseph University Beirut Lebanon

**Keywords:** epilepsy, quality of life, socioeconomic crisis

## Abstract

**Objective:**

The economic crisis in Lebanon, which began in 2019, has affected the healthcare system and patients' incomes. The aim of this study was to analyze the obstacles faced by people with epilepsy (PWE) during this crisis and to assess its impact on their quality of life.

**Methods:**

The method used was a cross‐sectional study conducted among PWE aged 18–65 years, who were asked to complete a comprehensive questionnaire covering sociodemographic aspects, clinical aspects, the impact of the economic crisis, and the QOLIE‐31 (version 1.0), validated in English and Arabic, which assesses the quality of life of PWE.

**Results:**

71 patients were included in the study with an average age of 35.2 years [23.5; 62.5] (53.5% were males). Their average QOLIE‐31 score was 50.3 (+/− 17.9). A significant proportion (71%) of patients reported difficulties during the crisis, with 25% reporting having had seizure‐related injuries in the years 2022–2023 and 36.6% reporting an increase in seizure frequency compared to that prior to 2020.

Moreover, many patients had to change (33.8%) or discontinue (18.3%) antiseizure medications, due to drug shortages, rising costs, and high gas prices. To mitigate these challenges, patients sought solutions such as obtaining medications from abroad (34%) or through donations (8%) or purchasing from the black market (8%).

Low quality of life was associated with unemployment, low education level, the presence of focal seizures with impaired awareness or generalized seizures, polytherapy, seizure‐related injuries, and medication changes during the economic crisis.

**Significance:**

These results highlight the considerable challenges faced by PWE in Lebanon during the economic crisis, emphasizing the negative effect of the crisis on their quality of life and seizure control.

**Plain Language Summary:**

This study analyzed the obstacles faced by 71 people with epilepsy during Lebanon's economic crisis and showed that many patients had to change (33.8%) or discontinue (18.3%) antiseizure medications, due to drug shortages, rising costs, and high gas prices. To mitigate these challenges, patients sought solutions such as obtaining medications from abroad (34%) or through donations (8%) or purchasing from the black market (8%). This affected their quality of life. In fact, low quality of life was associated with unemployment, low education level, focal seizures with impaired awareness or generalized seizures, polytherapy, seizure‐related injuries, and medication changes.

**Key Points**

- Epilepsy's chronic nature and diverse medical and psychosocial influences significantly affect the quality of life (QOL) of people with epilepsy (PWE).
- The Lebanese economic crisis forced people with epilepsy (PWE) to discontinue their medication due to shortages or high costs.
- To mitigate these challenges, patients obtained medications from abroad (34%) or through donations (8%) or from the black market (8%).
- This led to increased seizure‐related injuries and seizure frequency, and low QOL‐31 scores.
- Low QOL was associated with unemployment, low education level, seizures with impaired awareness, polytherapy, seizure‐related injuries, and drug changes.

## 1 INTRODUCTION

Lebanon is a Middle Eastern country, right on the Mediterranean coastline. It is a country that has experienced one of the most severe economic, financial, and political crises in its history since 2019. The situation has been exacerbated by the COVID‐19 pandemic and the Beirut port explosion in 2020. The collapse of the financial system led to severe restrictions on bank withdrawals, leaving many Lebanese people unable to access their savings. The economic slowdown continues to this day, albeit to a lesser degree, according to the latest World Bank report in 2023.^1^ To better illustrate the extent of the crisis, it should be mentioned that from 1997 until 2019, the Lebanese pound was pegged to the US dollar with a conversion rate of 1 US dollar = 1507.5 Lebanese pounds. In February 2023, it underwent devaluation and lost more than 98% of its initial value,^1^ reaching a depreciation of 59 times over the last 4 years with a conversion rate of 1 US dollar = 89 000 Lebanese pounds (December 28, 2023) on the black market.

As with any economic crisis, the health sector including epilepsy care has been significantly affected in recent years. Initially, from 2019 till 2021, due to government subsidies, most medications including antiseizure medications (ASM) remained affordable and could be bought from pharmacies at a low price in US dollars; however, this has led to smuggling and hoarding of these subsidies medications to sell them later at a higher price or sell them outside of the country for an increased profit, which worsened the shortage.^2^ Subsequently, in 2021, the decrease in the liquid USD dollars reserves of the Central Bank led to the Ministry of Health to remove subsidies for many drugs and cut the health sector spending by 40%. Therefore, medication prices began to rise and the pharmaceutical import plummet.^3^ Additionally, the government's plan to lift subsidies intensified the crisis as citizens, fearing a shortage, began hoarding them.

The increase in prices and the shortage of medications led people to buy them from neighboring countries such as Syria, Jordan, and Turkey, or from relatives abroad in Europe or North America.

Parallel to the drug shortage, there was a fuel shortage, followed by a considerable increase in fuel prices. Indeed, Lebanon is a country where public transportation is scarce or not well maintained, and most residents heavily rely on private cars or taxis. In addition, most university hospitals are concentrated in urban areas specifically Beirut. Residents of rural areas must travel 25–50 kilometers to reach the nearest hospitals, a tertiary care center, or a major pharmacy. This unequal distribution of healthcare facilities and services, along with the dependence on private cars or taxis for travel, made the fuel shortage crisis and rising fuel prices a significant obstacle for patients seeking medical help.

The main objective of this study is to analyze the obstacles faced by PWE in Lebanon during the latest economic crisis and to assess the impact of this crisis on their quality of life.

## 2 MATERIALS AND METHODS

### 2.1 Population and questionnaire

A cross‐sectional, descriptive study was conducted at the Epilepsy clinic of Hotel Dieu de France Hospital in Beirut, the university hospital of Saint Joseph University. This study was approved by the institution's ethics committee and aimed to evaluate the difficulties encountered by PWE during the economic crisis in Lebanon since 2019 and their impacts on their quality of life (QOL) using a questionnaire divided into three parts: The first part covered the sociodemographic information (including gender, age, marital status, region of residence, education level, employment status, socioeconomic status, dependent household members, presence of chronic disease) and disease‐related factors (seizure nature, frequency changes, prescribed treatment, adherence, specialist visits, injury/emergency room visits, comorbidities). The second part explored the difficulties encountered during the crisis such as difficulty accessing healthcare services, discontinuation or change of ASM, payment issues, missed medical appointments, financial strain, and self‐medication.

Whereas the third part consisted of the QOLIE‐31 (version 1.0) questionnaire, which is already, validated in English and Arabic,^4^, ^5^, ^6^ and assesses QOL specifically in PWE. The total score was calculated using the QOLIE‐31 scoring manual and ranges from 0 to 100, where 100 reflects excellent QOL. This questionnaire was available in English and Arabic, in electronic form (Google Forms), and on paper. It was sent via social networks (such as WhatsApp, Facebook,) to PWE through Epsilon Lebanon, a medical and social NGO for PWE, or filled out in person in clinics or at the hospital (Hôtel‐Dieu de France).

Recruitment began on June 1, 2023, and ended on December 28, 2023.

Patients with epilepsy diagnosed by a neurologist before 2019, aged 18–65 years, and residing in Lebanon were included in the study.

Patients under 18 years of age or suffering from intellectual, psychiatric, or cognitive disorders were excluded. Patients who were unable to complete the questionnaires themselves due to mental handicap were excluded.

### 2.2 Statistical analysis

For the statistical analysis of the data, IBM SPSS Statistics for Windows, version 26.0, was used.

The normality of the distribution was verified using a Q–Q plot. To evaluate the correlation between the QOLIE‐31 score and the number of individuals dependent on monthly income, a Spearman correlation was applied due to the nonparametric nature of the distribution. To determine whether there was a significant difference between the overall QOLIE‐31 score and the QOLIE‐31 subsets in terms of the qualitative sociodemographic variables, clinical characteristics, and obstacles encountered during the economic crisis, Student's t test or the Mann–Whitney test was used (depending on the normality of the distribution of values) for only two groups (e.g., 0, recorded as yes, and 1, recorded as no), or ANOVA or the Kruskal–Wallis test was applied (depending on the normality of the distribution of values) for three or more groups. A difference in QOL was considered significant if the *p* value was less than 0.5.

## 3 RESULTS

### 3.1 Descriptive analysis

#### 3.1.1 Sociodemographic and disease‐related characteristics

Our population consisted of 71 patients, 53.5% male and 46.5% female, with an average age of 35.2 years [23.5; 62.5]. Thirty‐eight individuals (53.3%) were married, 30 (43.3%) were single, and 3 (4.2%) were divorced.

The sociodemographic and clinical characteristics of the patients with epilepsy are represented in Table 1.

**TABLE 1:** Sociodemographic and clinical characteristics of the participants.

Sociodemographic characteristics	% or mean (SD)
Total number of participants	71
Age	35.2
Place of residence	
Mount‐Lebanon	31.0%
Beirut	23.9%
Keserwen‐Jbeil‐North regions	21.1%
Baalbak‐Hermel‐Bekaa regions	12.7%
South regions	11.3%
Level of education	
University	59.2%
College	12.7%
High School	21.1%
Less than High School	7.0%
Employment	
Yes	53.5%
No	46.5%
Total household monthly income	
<100 USD	29.6%
100–500 USD	31.0%
>500 USD	39.4%
Number of individuals dependent on this income	3.3 (+/− 1.57)

Clinical characteristics	
Seizure type	
Absence	14.8
Myoclonic	9.8
Focal (aware or impaired awareness)	33.6
Generalized (Atonic, tonic–clonic, unknown)	25.4
Unclassified	16.4
ASM prescribed	
Sodium Valproate	12.3
Carbamazepine	11.6
Oxcarbazepine	4.3
Phenytoin	1.4
Gabapentin	0.7
Phenobarbital	2.2
Pregabalin	1.4
Lamotrigine	16.7
Levetiracetam	33.3
Topiramate	2.2
Mysoline	0.7
Lacosamide	5.8
Other	7.2
Number of seizures in the preceding 12 months	
0	32.4
1	19.7
2–10	22.5
11–20	15.5
21–50	7.0
>50	2.8
Adherence to ASM	
I always remember to take my medication on time, as prescribed	64.8
I am sometimes forgetful about taking it	22.5
I often have trouble taking my medication	2.8
I don't see why I should take it	2.8
None of the above	5.6
N/A	1.4

Abbreviations: ASM, Antiseizure medication; N/A, not applicable; USD, United States Dollar.

The majority of our participants consulted a health professional for their epilepsy 1–2 times a year (53.5%), followed by those who did so 3–4 times a year (26.8%). A smaller percentage of participants (14.1%) reported visiting their doctor 5 times a year or more or not visiting them at all (5.6%).

More than half of our population, 54.9% (39 patients), reported using an association of 2 or more ASM.

36.6% of patients reported an increase in seizure frequency compared to that in the years before 2020, and 25.4% (18 individuals) of participants declared having had injuries or needed emergency care due to epilepsy in the past 12 months.

#### 3.1.2 Impact of the economic crisis

**FIGURE 1:**
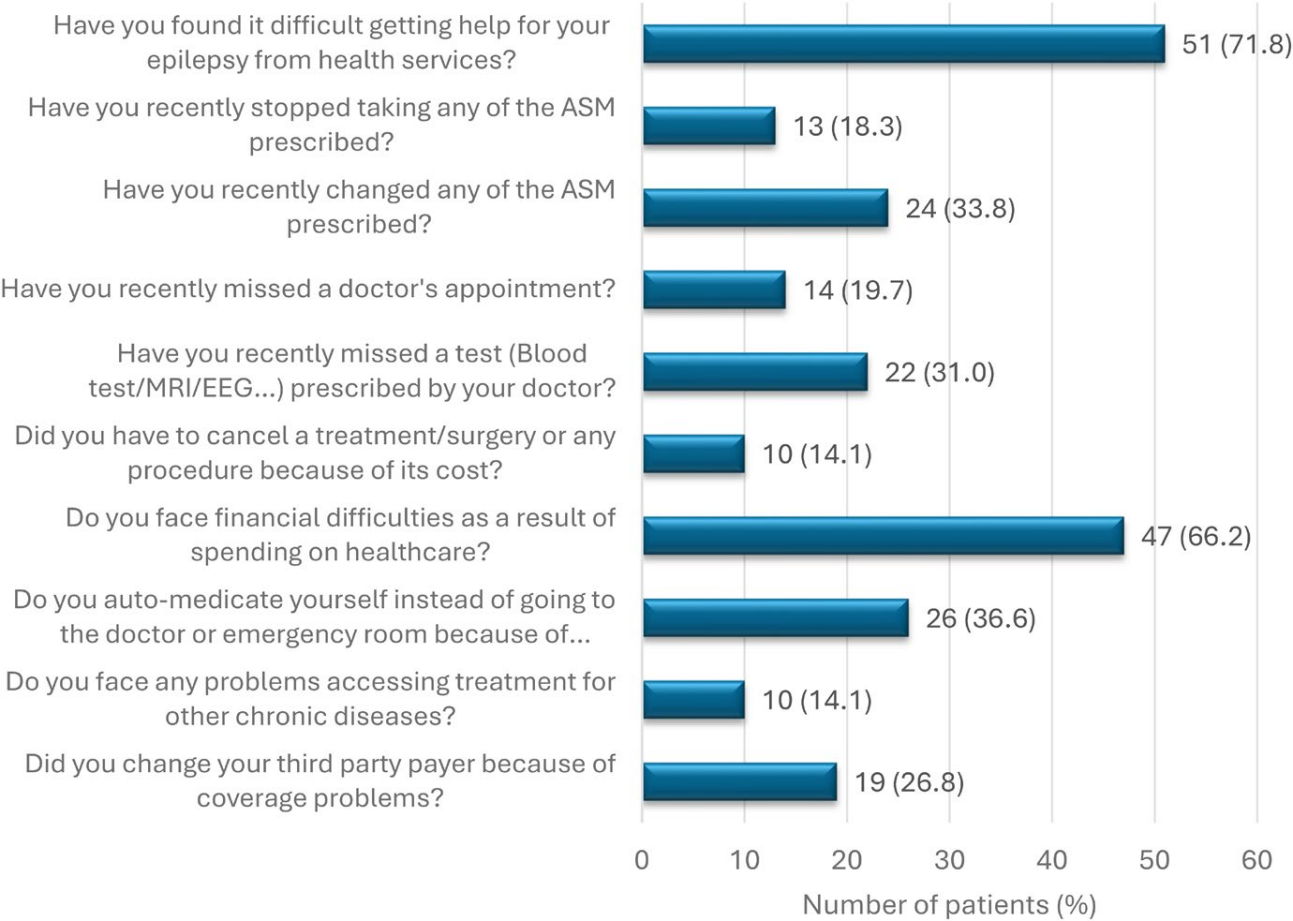
Responses to questions regarding the impact of the economic crisis on PWE. ASM, Antiseizure medications; PWE, People with epilepsy.

**FIGURE 2:**
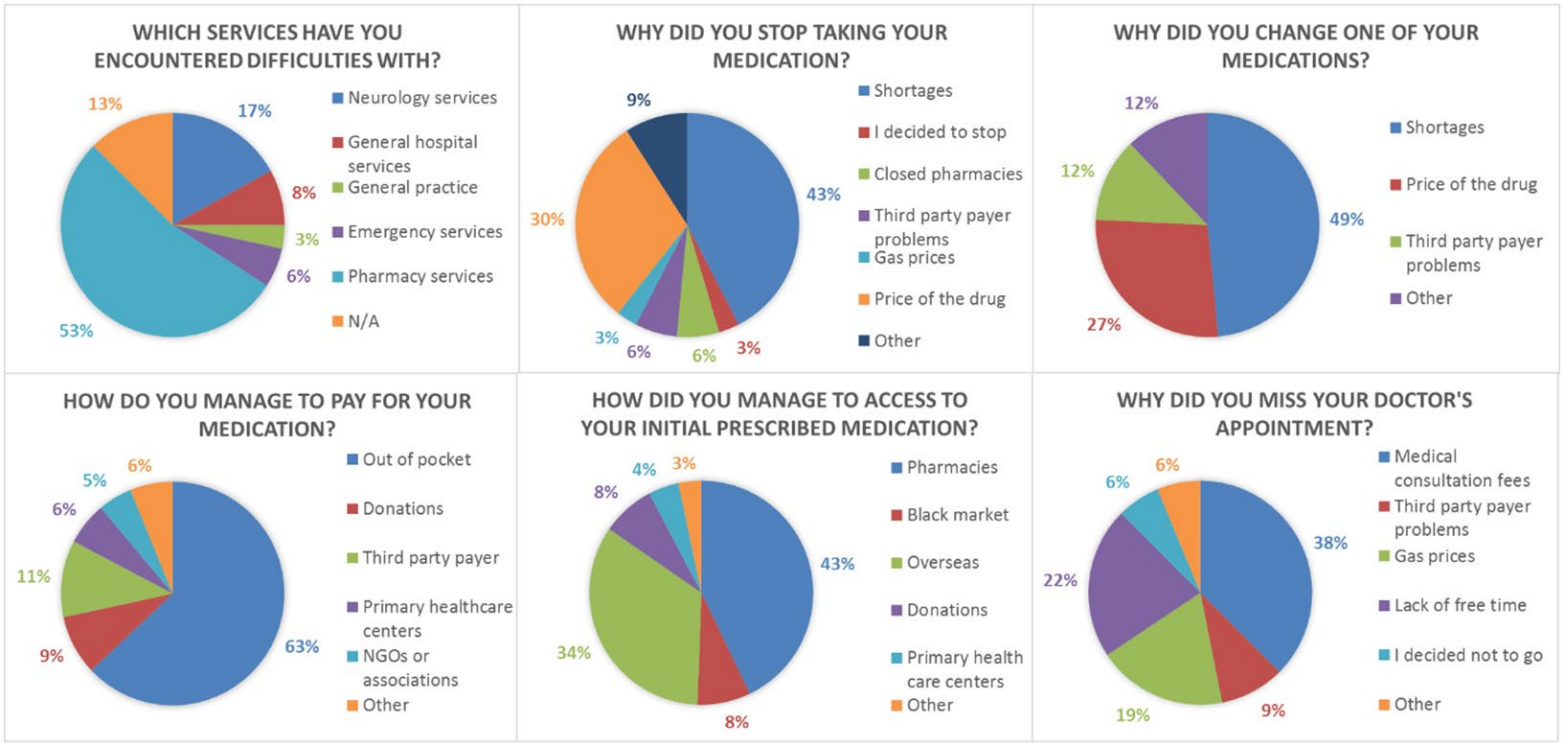
Pie charts showing the breakdown of responses to the questions regarding the impact of the economic crisis. NGO, Non‐governmental organization.

The number (percentage) of individuals who responded “Yes” to questions about the economic crisis and the distribution of their answers are represented in Figures 1 and 2, respectively.

Fifty‐six individuals (78.9%) were able to access the initially prescribed medication.

Among the 10 individuals who were recommended to undergo surgical intervention for their epilepsy, 3 patients refused the intervention, two of them (66.6%) due to fear of surgery, and one of them (33.3%) due to the high cost of the surgery.

In our population, 19.7% (14 individuals) suffered from chronic diseases that required medical treatment, of which 71.4% (10 patients) reported having difficulties accessing their treatments.

Among those who missed prescribed exams by their doctor, the main reason appears to be related to the high cost (61.1% of participants). Third‐party payer coverage problems and fuel prices each represent 16.7%.

In terms of the perception of access to the healthcare system, 56.3% of participants believed the situation was worse in 2023 than in previous years, while 29.6% thought it was the same. Only 14.1% considered that access to healthcare had improved.

#### 3.1.3 QOL measured by the QOLIE‐31

The average and standard deviation of the overall QOLIE‐31 score and that of each subset are presented in Table 2.

**TABLE 2:** Mean and standard deviation of the overall score of the QOLIE‐31 and its subsets.

	Seizure worry	Overall QOL	Emotional well‐being	Energy Fatigue	Cognitive	Medication Side effects	Social function	Overall score
Mean	40.0	53.9	47.6	45.0	48.4	47.2	59.9	50.3
Standard deviation (SD)	23.4	20.9	20.2	21.0	24.2	31.7	26.1	17.9

Abbreviation: QOL, quality of life.

### 3.2 Comparative analysis

The results of the comparison of the overall QOLIE‐31 score and its subsets according to the demographic and clinical characteristics of the patients are represented in Table 3, and the comparisons according to the patients' responses to the impact of the economic crisis are shown in Table 4.

**TABLE 3:** Correlation of the overall and subsets score of the QOLIE‐31 and the demographic and clinical characteristics of the patients.

		Overall score mean (SD)	Seizure worry mean (SD)	Overall QOL mean (SD)	Emotional mean (SD)	Energy mean (SD)	Cognitive mean (SD)	Medication effects mean (SD)	Social mean (SD)
Gender	Male	51.8 (18.4)	44.9 (23.7)	51.0 (22.6)	47.6 (21.9)	48.4 (21.6)	52.5 (22.1)	50.3 (32.0)	59.6 (25.9)
	Female	48.3 (17.3)	34.4 (22.2)	55.3 (18.9)	47.5 (18.4)	41.2 (20.0)	43.7 (25.9)	43.7 (31.5)	60.3 (26.7)
	*p* value	0.41	0.06	0.4	0.99	0.15	0.13	0.39	0.9
Work	Yes	54.5 (14.9)	42.7 22.5)	56.0 (15.9)	52.6 (18.8)	50.3 (15.6)	52.4 (22.1)	50.7 (31.2)	64.8 (23.1)
	No	45.3 (19.9)	36.9 (24.5)	49.5 (25.3)	41.7 (20.4)	39.0 (24.8)	43.8 (26.0)	43.1 (32.3)	54.3 (28.5)
	*p* value	0.031*	0.3	0.21	0.02*	0.03*	0.14	0.32	0.09
Education level	Less than High School	40.8 (20.1)	36.8 (22.7)	58.0 (29.2)	45.6 (23.3)	42.0 (27.1)	33.7 (12.1)	47.2 (38.3)	35.0 (30.7)
	High School	41.3 (18.4)	29.0 (20.4)	39.5 (26.9)	40.5 (18.6)	36.0 (22.3)	40.6 (24.0)	43.9 (29.0)	51.1 (25.3)
	College	48.6 (7.2)	48.7 (24.6)	45.0 (16.2)	46.2 (15.4)	43.3 (11.5)	49.1 (14.6)	30.6 (33.6)	57.4 (17.0)
	University	54.9 (17.8)	42.5 (23.6)	58.9 (15.7)	50.6 (21.3)	49.0 (20.1)	52.8 (26.1)	52.0 (31.3)	66.6 (25.4)
	*p* value	0.039*	0.16	0.008*	0.42	0.22	0.19	0.313	0.024*
Total household income	< 100 USD	46.2 (18.3)	31.7 (24.0)	46.1 (23.8)	41.1 (18.0)	47.0 (21.6)	47.1 (23.8)	46.7 (29.4)	53.9 (27.2)
	100–500 USD	48.0 (16.6)	36.5 (19.1)	50.0 (19.2)	46.7 (19.3)	39.0 (19.3)	48.3 (24.8)	45.5 (33.4)	57.0 (26.3)
	>500 USD	55.0 (17.9)	49.0 (23.8)	60.4 (18.1)	53.0 (21.6)	48.3 (21.6)	49.5 (24.9)	49.0 (33.1)	66.7 (24.4)
	*p* value	0.187	0.02*	0.04*	0.12	0.27	0.95	0.92	0.2
Focal seizures with impaired awareness	Yes	40.7 (18.2)	27.1 (18.3)	48.4 (21.8)	36.0 (19.0)	37.3 (25.8)	36.5 (26.1)	37.8 (34.7)	52.0 (29.3)
	No	53.0 (16.9)	43.8 (23.6)	54.3 (20.7)	50.9 (19.4)	47.3 (19.1)	51.9 (22.7)	49.9 (30.6)	62.2 (24.9)
	*p* value	0.015*	0.01*	0.33	0.008*	0.09	0.02*	0.18	0.17
Generalized seizures (Atonic, tonic–clonic, unknown)	Yes	38.3 (16.9)	21.8 (21.2)	44.8 (14.6)	35.2 (16.0)	30.5 (14.4)	42.4 (27.3)	24.4 (29.5)	43.7 (31.3)
	No	52.2 (17.4)	43.0 (22.6)	54.3 (21.6)	49.6 (20.2)	47.4 (21.1)	49.4 (23.8)	50.9 (30.9)	62.6 (24.4)
	*p* value	0.02*	0.007*	0.18	0.04*	0.02*	0.4	0.01*	0.03*
Injuries or need for emergency care in the last 12 months	Yes	41.6 (16.4)	29.8 (20.2)	46.1 (23.7)	40.2 (22.4)	37.1 (19.6)	37.7 (17.9)	34.4 (27.2)	52.9 (26.6)
	No	53.1 (14.5)	43.5 (23.6)	55.3 (19.6)	50.1 (19.0)	47.7 (21.0)	52.1 (25.1)	51.5 (32.2)	62.3 (25.7)
	*p* value	0.017*	0.03*	0.11	0.07	0.06	0.03*	0.047*	0.18
Number of ASM	1	55.3 (16.5)	40.2 (23.7)	55.7 (20.4)	49.8 (18.0)	47.4 (21.7)	54.4 (26.3)	56.9 (26.4)	70.2 (22.4)
	2 or more	46.3 (18.0)	39.9 (23.6)	50.8 (21.3)	45.8 (21.8)	43.3 (20.7)	43.7 (21.7)	39.7 (33.7)	52.0 (26.2)
	*p* value	0.034*	0.96	0.33	0.41	0.42	0.07	0.023*	0.003*

Abbreviations: ASM, Antiseizure medications; QOL, quality of life.

*p value <0.05.

**TABLE 4:** Correlation between the overall and subsets score of the QOLIE‐31 and the questions related to the impact of the economic crisis.

		Overall score mean (SD)	Seizure worry mean (SD)	Overall QOL mean (SD)	Emotional mean (SD)	Energy mean (SD)	Cognitive mean (SD)	Medication effects mean (SD)	Social mean (SD)
Difficulty getting help for your epilepsy from health services	Yes	48.5 (18.2)	37.0 (23.4)	53.1 (21.5)	45.2 (19.4)	42.3 (20.8)	47.7 (24.4)	45.6 (32.1)	57.3 (26.8)
	No	54.5 (16.7)	47.7 (22.2)	52.6 (19.9)	53.4 (21.5)	51.9 (20.9)	50.2 (24.2)	51.3 (31.2)	66.6 (23.6)
	*p* value	0.21	0.08	0.93	0.13	0.09	0.69	0.51	0.18
Stopped any of the ASM	Yes	46.7 (20.4)	41.9 (28.8)	47.9 (18.8)	52.9 (18.1)	43.2 (19.2)	40.7 (26.1)	45.7 (38.6)	53.3 (27.5)
	No	51.0 (17.3)	39.6 (22.3)	54.1 (21.4)	46.3 (20.6)	45.5 (21.6)	50.1 (23.7)	47.6 (30.4)	61.4 (25.8)
	*p* value	0.43	0.7	0.34	0.29	0.73	0.21	0.85	0.32
Changed any of the ASM	Yes	43.9 (17.5)	33.8 (22.3)	45.5 (23.4)	43.8 (20.1)	40.3 (21.8)	40.2 (22.2)	37.9 (31.8)	54.3 (25.8)
	No	53.5 (17.3)	43.2 (23.6)	56.7 (18.6)	49.4 (20.2)	47.5 (20.4)	52.6 (24.4)	51.9 (30.9)	62.8 (26.0)
	*p* value	0.03*	0.11	0.03*	0.27	0.17	0.04*	0.08	0.19
Access to your initial prescribed medications	Yes	50.2 (18.1)	39.1 (23.9)	52.7 (21.6)	47.4 (19.9)	44.4 (21.5)	48.8 (25.6)	49.6 (31.9)	59.9 (26.1)
	No	50.4 (17.5)	43.3 (21.8)	54.0 (18.4)	48.0 (22.1)	47.6 (19.6)	47.1 (18.9)	38.5 (30.4)	60.0 (26.9)
	*p* value	0.97	0.55	0.83	0.93	0.61	0.82	0.23	0.99
Missed a prescribed test	Yes	47.9 (17.7)	37.0 (25.1)	45.9 (23.3)	38.5 (19.3)	36.7 (20.1)	53.0 (25.6)	44.2 (26.7)	60.2 (27.6)
	No	50.8 (18.0)	40.7 (23.2)	54.7 (20.2)	49.7 (19.9)	47.1 (21.0)	47.2 (23.9)	47.9 (33.0)	59.9 (25.9)
	*p* value	0.59	0.59	0.16	0.06	0.10	0.43	0.69	0.96
Missed a doctor's appointment	Yes	47.8 (15.5)	38.7 (21.6)	49.3 (23.8)	38.9 (18.5)	38.6 (19.5)	47.1 (24.2)	46.5 (27.6)	62.7 (25.6)
	No	51.3 (18.9)	40.6 (24.4)	54.6 (19.5)	51.4 (19.9)	47.9 (21.8)	49.0 (24.4)	47.6 (33.7)	58.7 (26.5)
	*p* value	0.44	0.74	0.33	0.02*	0.09	0.77	0.89	0.55
Financial difficulty as a result of spending on healthcare	Yes	47.7 (18.2)	36.8 (22.4)	48.5 (22.3)	44.6 (20.3)	42.1 (21.3)	47.8 (24.4)	45.4 (29.5)	57.0 (26.8)
	No	55.2 (16.4)	46.2 (24.6)	61.7 (14.7)	53.4 (19.11)	50.8 (19.7)	49.7 (24.3)	50.8 (36.2)	65.6 (54.2)
	*p* value	0.09	0.11	0.004*	0.08	0.1	0.76	0.53	0.19

Abbreviations: ASM, antiseizure medication; QOL, quality of life.

*p value <0.05.

The results indicated that certain factors, such as sex, age, total household income, place of residence, type of epileptic seizure (other than focal seizures with altered consciousness and tonic–clonic seizures), number of annual consultations with a health specialist, and adherence to ASM, did not significantly affect the overall QOLIE‐31 score.

No significant correlation was detected between the number of individuals dependent on household income and the overall QOLIE‐31 score. However, a significant negative correlation (−0.296, *p* value 0.018) was observed with the energy/fatigue subset.

## 4 DISCUSSION

The results of this study provide an important overview of the challenges faced by PWE in Lebanon during the economic crisis.

### 4.1 QOL of PWE in Lebanon before and after 2020

Our average overall QOLIE score of 50.3 +/− 17.9, lower than the global average QOLIE‐31 score among 7255 patients from 31 countries is 59.8 +/− 8.0.^7^ It should be noted that this study was published in 2016 before the COVID‐19 pandemic.

In addition, the score obtained in our study is clearly lower than that of a similar study conducted in Lebanon (64.69 +/− 19.3) published in 2022 but whose responses were recorded in 2018, before the economic crisis and the COVID‐19 crisis that devastated the country in 2019.^8^ The decrease in the average overall QOLIE‐31 score in Lebanon could be linked to the economic crisis or the COVID‐19 pandemic.

### 4.2 Sociodemographic and disease‐related characteristics and their relationship with the economic crisis and QOL of PWE

Highlighting that 36.6% of patients reported increased seizure frequency compared to pre‐2020 levels is crucial. This finding may help explain the decline in patients' QOL observed between our study and one conducted in Lebanon in 2022. Increased seizure frequency is a significant predictor of poor QOL in epileptic patients.^9^, ^10^, ^11^ Notably, the QOLIE‐31 subgroup showed the lowest score in the subset related to seizure concerns and the highest in social function, consistent with findings from a 2022 study in Lebanon. This increased concern may be influenced by economic constraints and limited access to medical services, affecting disease management effectiveness.

In our population, unemployment incidence was 46.5%, significantly associated with reduced QOL, and lower emotional and energy subscales of QOLIE‐31, consistent with findings in Lebanon,^8^, ^12^ and Iraq.^13^ Employment positively affects QOL, potentially due to monthly income, social integration, self‐confidence, and access to medical support.

A high rate of unemployment among patients with epilepsy in Lebanon, compared to 13.76% of the general population unemployed in Lebanon in 2024,^14^ can be first explained by their medical condition (seizures and cognitive dysfunction). In addition, the economic crisis in Lebanon has severely limited job opportunities to people with and without epilepsy, and the stigma associated with epilepsy further exacerbates the challenges these individuals face in finding and securing employment.

Despite economic challenges, our unemployment prevalence was lower than previous Lebanese studies, possibly suggesting patients' motivation to work. It could also be explained by our choice to exclude patients with severe cognitive and intellectual disorders. This can increase medication adherence^15^; however, it may lead to anxiety regarding seizures affecting work abilities, particularly as 70.3% support families composed of 2–4 people and rely on stable employment.

Surprisingly, our study revealed no significant difference in the overall QOLIE‐31 score among the three income groups. This finding contrasts with the results of the study conducted by Abou Zeki et al.^8^ and that in Iraq^13^ but is in line with the findings obtained in Malaysia.^16^ A significant difference was observed in the subsets of “concern about seizures” and “overall QOL.” This disparity could be explained by a low number of participants in our study. Another explanation could be that the participants in this study had good medical follow‐up and received good social or other support regardless of their monthly income, contributing to better QOL in certain aspects. Hence, it is important to recognize the role of NGOs in Lebanon and different patient support groups (such as EPSILON association which is based in our institution) in providing medical and social support to people with epilepsy.

Individuals who reached the highest level of education, namely, university, showed a significant improvement in their QOL. This observation, comparable to the results of Mroueh et al. (2020) in Lebanon^12^ or a study in Malaysia^16^ but contradictory to those of Abou Zeki et al.^8^ or a study in Iraq,^13^ could be explained by a greater support network from their peers, an increased professional and financial stability and an in‐depth understanding of their illness.^17^ Patients with intellectual limitations were excluded from the study, which could introduce some selection bias. This exclusion as mentioned in our methodology was made deliberately due to the inability of these individuals to complete the questionnaire themselves.

The use of multiple ASM has been associated with reduced QOL, a finding consistent with several previous studies.^9^, ^10^ Indeed, polytherapy can increase the risk of side effects and drug interactions, and the risk of forgetfulness and nonadherence.^18^ Moreover, during the crisis in Lebanon, patients had major difficulties in procuring medications, leading to a heavier financial burden and, consequently, a reduced QOL.

A significant number of patients (25.4%, 18 individuals) reported injuries or the need for emergency care due to epilepsy in the past 12 months, which raises concerns about safety and seizure management in this population. According to a 1990 study, the annual risk of an epileptic person visiting the emergency room due to a seizure is 5%,^19^ but there is a discrepancy in this rate, varying between 1.2% per year in Norway, 9% and 14% in two studies in Canada.^19^, ^20^, ^21^ In addition, our study revealed an association between the presence of injuries or the need for emergency care and lower scores on the QOLIE‐31. The physical and emotional consequences of injuries and emergencies can lead to a significant deterioration in QOL and increase in mortality.^3^, ^22^

Regarding clinical follow‐up, 53.5% of participants consulted a health professional for epilepsy 1–2 times a year. This frequency may be influenced by factors such as the COVID‐19 pandemic, fuel shortages, and high medical consultation costs. Despite 19% of patients missing appointments due to financial constraints and time limitations, most maintained regular contact with their physician via telephone services, benefiting from the advantages of telemedicine, a concept developed during the pandemic.^23^, ^24^ Telemedicine adaptation helps overcome barriers like availability, costs, and mobility, ensuring continuous follow‐up despite economic and logistical challenges.

### 4.3 Magnitude of the economic crisis

To date, the economic crisis in Lebanon has reached alarming proportions, marked by a drastic devaluation of the Lebanese pound. With 80% of drugs imported and 63% of patients having to pay for their medications out of pocket, citizens sought alternatives, including sourcing from neighboring countries or receiving medications from relatives abroad, highlighting the deterioration of access to healthcare in Lebanon.^2^, ^3^

The results of our study shed light on the significant impact of the economic crisis in Lebanon on epilepsy management and the overall health of patients. First, the alarming finding that 71.8% of respondents encountered difficulties during the crisis, with over half reporting problems in pharmacy services, underscores the extent of obstacles in accessing essential medications. The consequences of the drug shortage are evident, with 18% of participants having stopped their ASM and 33.8% having changed their treatment, mainly due to limited availability and high costs.

Additionally, the high prevalence of 31% of patients missing prescribed medical exams due to high costs indicates a deterioration of essential follow‐up care. A small study conducted between 2020 and 2022 on pregnant women on ASM showed that only 5 out of 23 women were followed up with blood level tests.^25^

Financial difficulties are also a major problem, with 66.2% reporting budget constraints related to health expenses, which led to self‐medication by 36.6% of participants. Finally, the correlation between difficulties accessing medications for other chronic diseases (71%) and the economic crisis underlines the widespread impact of the crisis on the entire health system in Lebanon, a result in line with studies conducted in Lebanon after the crisis on several chronic diseases (COPD, cancer, antihypertensive).^3^, ^8^, ^12^, ^13^

It is notable that our study found a significant decrease in the QOLIE‐31 score among patients who changed anti‐seizure medication (ASM) compared to those who did not. Several studies offer conflicting findings; one indicates no significant impact on QOL after transitioning from branded to generic Levetiracetam,^26^ while another report increased seizure frequency and side effects post‐switch^27^ but the social conditions of our population are different.

Moreover, significant decrease in the emotional subset score among patients who missed a doctor's appointment highlights the impact of regular medical follow‐up on the emotional dimension of the QOL. The absence of medical visits can generate worries, anxiety, and uncertainties in patients, thus contributing to a deterioration of their emotional well‐being.

### 4.4 PWE during past difficult times

Our results were comparable to that found in other countries that were affected by an economical or natural disaster such as Syria,^28^ Yemen^29^ or Iran.^29^ In Iran, secondary to economic sanctions, more than a third of patients had difficulty obtaining prescribed ASM and 15% of patients reported that their medications were completely inaccessible.^29^ This was similarly described in Ukraine due to the war.^30^, ^31^, ^32^, ^33^ In addition, a study published in Sri Lanka comparing health outcome of patients with epilepsy before and after the economic crisis reported an increase in the average number of seizures and breakthrough seizures, as well as an increase in the number of emergency admissions or psychological distress in PWE, their caregivers and care providers after the crisis. The ASM shortages were the main challenges patients faced during these times. 90% of their population reported having difficulties finding the prescribed medication due to shortages, high cost, or transportation issues.^34^ The economic crisis in Sri Lanka was due to multiple factors including the COVID‐19 pandemic and political and economic factors, which is similar to our situation.

In fact, the COVID‐19 pandemic impacted PWE in multiple countries around the world and limited their access to healthcare significantly. An international survey published in 2021 showed that a considerable proportion of patients had difficulty obtaining prescribed antiepileptic drugs (30%) due to a shortage of drugs in most cases, thus observing an increase in the frequency of seizures (30%), and an increased frequency of psychiatric comorbidity (anxiety 72.5%‐depression 39.1).^35^ The reflection of this health crisis is also well described by a Serbian study, which, using the QOLIE‐31, showed that patients had a lower quality of life during the pandemic compared to before it (*p* < 0.001), that was associated with fear of seizures and fear of reduced household income. Moreover, their QOLIE‐31 score during the COVID‐19 pandemic was 64.4 (+/− 14.6) which is significantly higher than the results of our study during the economic crisis in Lebanon.^36^

### 4.5 Coping strategies of PWE at times of crisis

The result indicating that 78.9% of patients were able to access the initially prescribed medication despite the challenges posed by the economic crisis in Lebanon reflects several dynamics. First, the reliance on pharmacies as the primary source of supply underlines the importance of the pharmaceutical sector in maintaining access to medications. Additionally, the significant contribution of purchases abroad reinforces the essential role of the Lebanese diaspora and family and peer support networks, demonstrating solidarity and mutual aid within the community. The ability of the product (boxes and tablets) to be easily transported and storable facilitated the procurement of medications from abroad, unlike some drugs that require specific storage conditions, such as anticancer drugs or some multiple sclerosis treatments. Moreover, the use of the black market and donations highlights the adaptation strategies implemented by patients to overcome financial obstacles and shortages.

### 4.6 Limitations

This study has limitations worth noting. Its cross‐sectional design restricts the establishment of causal relationships between variables, highlighting the need for longitudinal studies for deeper insights into patients' conditions over time.

Additionally, reliance on self‐reported data, especially regarding seizure frequency, raises concerns about accuracy due to subjective interpretation and potential response and recall biases. Despite efforts to clarify the questions, it remains possible that participants did not uniformly interpret the questions about the frequency and types of seizures, so we did not include this in the comparative analysis.

On the other hand, the small sample size of 71 might limit the identification of significant correlations.

Lastly, even though participants came from all Lebanese regions, most were recruited from one center in Beirut, and this could have overestimated the QOL scores preventing generalization to the whole epilepsy community of Lebanon.

## 5 CONCLUSION

Our study identified multiple obstacles faced by patients with epilepsy in the settings of the difficult socioeconomic context.

Indeed, it showed that a large proportion of patients had to change or stop their ASM, cancel necessary tests or surgeries, or miss their consultations with their neurologist for several reasons, particularly the high costs of services and medications, drug shortages, and gas prices.

In conclusion, this study underscores the necessity of adjusting clinical and social strategies to address the multifaceted needs of this vulnerable population by employing a comprehensive and holistic approach to ensure optimal care and quality of life for PWE.

To prevent similar consequences in the future, governmental and legislative actions are necessary. First, robust public health policies must be implemented to secure continuous accessibility to essential medications by limiting smuggling and ensuring affordable prices. Second, an integrated initiative at the economic, public health, and social levels is essential to create a good infrastructure that meets the needs of this population, even in times of crisis or unforeseen events.

## CONFLICT OF INTEREST STATEMENT

None of the authors has any conflict of interest to disclose.

## ETHICS STATEMENT

We confirm that we have read the Journal's position on issue involved in ethical publication and affirm that this report is consistent with those guidelines.

## Data Availability

The data that support the findings of this study are available on request from the corresponding author. The data are not publicly available due to privacy or ethical restrictions.
